# Extraction and Isolation of Two Polysaccharides from *Chloranthus japonicus* Sieb. and Evaluation of Their Anti-Gastric Cancer Activities

**DOI:** 10.3390/molecules29092043

**Published:** 2024-04-29

**Authors:** Ju Liu, Wenfeng Li, Lu Jin, Yingchao Wang, Xinjun Xu, Enyao Ma, Depo Yang, Zhimin Zhao

**Affiliations:** 1 School of Pharmaceutical Sciences, Sun Yat-sen University, Guangzhou 510006, China; 2 Department of Drug Discovery, Guangzhou Caizhilin Pharmaceutical Co., Ltd., Guangzhou 510360, China

**Keywords:** *Chloranthus japonicus* Sieb., polysaccharide, structure features, anti-gastric cancer

## Abstract

Two unreported heteropolysaccharides, denoted as YCJP–1 and YCJP–2, were isolated from the herbs of *Chloranthus japonicus*. YCJP–1 was a heteropolysaccharide composed of glucose, galactose, arabinose, mannose, rhamnose, and a minor proportion of uronic acids, with the molecular weight mainly distributed in the 74,475–228,443 Da range. YCJP–2 was mainly composed of glucose, mannose, and galactose, with the molecular weights ranging from 848 to 5810 Da. To further evaluate the anti-gastric cancer effects of *C. japonicus*, the inhibitory effects of the crude polysaccharide (YCJP) and the purified polysaccharides (YCJP–1 and YCJP–2) were determined using a CCK-8 assay and colon-forming assay on MGC-803 and AGS gastric cancer cell lines. Our results showed that YCJP, YCJP–1, and YCJP–2 possess prominent inhibitory effects on the proliferation of MGC-803 and AGS cells, and the AGS cell was more sensitive to YCJP, YCJP–1, and YCJP–2. Moreover, YCJP–2 demonstrated superior anti-gastric cancer effects compared to YCJP–1. This could potentially be attributed to YCJP–2’s higher glucose content and narrower molecular weight distribution.

## 1. Introduction

*Chloranthus japonicus* Sieb. (*C. japonicus*) is a perennial herbaceous plant belonging to the Chloranthus family; it is mainly distributed in the northeast of China, the far east of Russian, Japan, and Korea [1]. *C. japonicus* was traditionally used for the treatment of various diseases, including cough, due to the wind-cold pathogen, traumatic injury, and snakebites, etc. [2]. Modern pharmacological research suggests that *C. japonicus* possesses various beneficial properties, such as anti-tumor, anti-inflammatory, antiviral, and antibacterial effects [3,4]. The active substances corresponding to the benefits of *C. japonicus* are reported to be flavonoids, saponins, lignans, and terpenoids [4,5,6,7,8]. Recently, although recent studies have been conducted on *C. japonicus*, the majority of research has focused on the structures and biological activities of small-molecule substances. There are relatively few reports on biomolecules [9].

Polysaccharides are a kind of biomacromolecule with a broad spectrum of biological and pharmacological activity, including anti-tumor, anti-inflammatory, anti-oxidative, and immunomodulatory activity [10]. Among these pharmacological activities, the anti-tumor activity is the most significant and has attracted increasing attention in recent years [11]. For instance, the polysaccharide from mushroom, known as lentinan, has been used in the clinical treatment of cancer in China and Japan for many years [12]. Moreover, polysaccharides such as genistein, *Grifola frondosa* polysaccharide, *Schizophyllum commune* polysaccharide, and *Trametes versicolor* polysaccharide were also proven to possess prominent anti-tumor activity and have been widely used in clinical trials [11]. Therefore, searching for potential anti-tumor candidate drugs from natural active polysaccharides has become an effective way to develop new anti-tumor drugs. *C. japonicus* has been confirmed to possess obvious anti-tumor activity [2,9]. However, the active substances corresponding to the anti-tumor activity of *C. japonicus* are still unclear. *C. japonicus* polysaccharides (CJP) are one of the bioactive components of *C. japonicus*; nevertheless, most of the studies mainly focused on small-molecule substances, with very limited studies relating to the structural features or anti-tumor activities of *C. japonicus* polysaccharides.

Thus, our study aimed to obtain purified bioactive polysaccharides from *C. japonicus*. The structure features of the obtained polysaccharides were preliminarily characterized using high-performance liquid gel permeation chromatography (HPGPC), ultraviolet visible (UV-Vis) spectrophotometry, Fourier transform infrared (FT-IR) spectroscopy, monosaccharide composition analysis, and scanning electron microscopy (SEM). Furthermore, the anti-gastric cancer activities of YCJP, YCJP–1, and YCJP–2 were determined using a CCK-8 and colon-forming assay.

## 2. Results and Discussion

### 2.1. Preparation of YCJP

YCJP was prepared using the water extraction and alcohol precipitation method. The yield of YCJP was calculated to be 1.64%. In addition, the contents of polysaccharide, protein, and uronic acid in YCJP were calculated to be 80.74%, 1.35%, and 16.27%, respectively.

### 2.2. Isolation of YCJP–1 and YCJP–2

**Figure 1:**
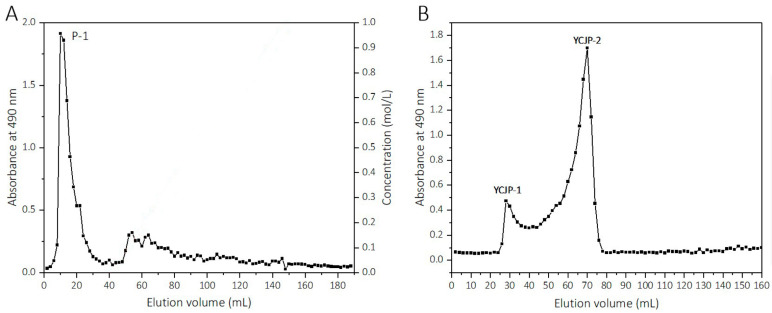
Elution curves of YCJP on DEAE-FF column and P-1 on Sepharose CL-6B column. (**A**) Elution curve of YCJP on DEAE-FF column. (**B**) Elution curve of P-1 on Sepharose CL-6B column.

YCJP was further fractionated using a DEAE-FF column and Sepharose CL-6B column, and two polysaccharides, YCJP–1 and YCJP–2, were obtained. The eluted curve of YCJP on the DEAE-FF column is shown in Figure 1A. The eluents that were eluted with distilled water were collected and labelled as P-1. After that, P-1 was further fractionated on a Sepharose CL-6B column, and two eluents was obtained, which were labelled as YCJP–1 and YCJP–2. The eluted curve of P-1 on the Sepharose CL-6B column are shown in Figure 1B.

The recoveries of YCJP–1 and YCJP–2 were calculated to be 1.02% and 6.01% based on the weight of YJCP, respectively. In addition, the polysaccharide contents of YCJP–1 and YCJP–2 were calculated to be 97.47% and 98.34%, and the protein contents of YCJP–1 and YCJP–2 were calculated to be 1.27% and 1.01%, respectively.

### 2.3. Structural Features of YCJP–1 and YCJP–2

#### 2.3.1. Relative Molecular Weight Distribution

**Figure 2:**
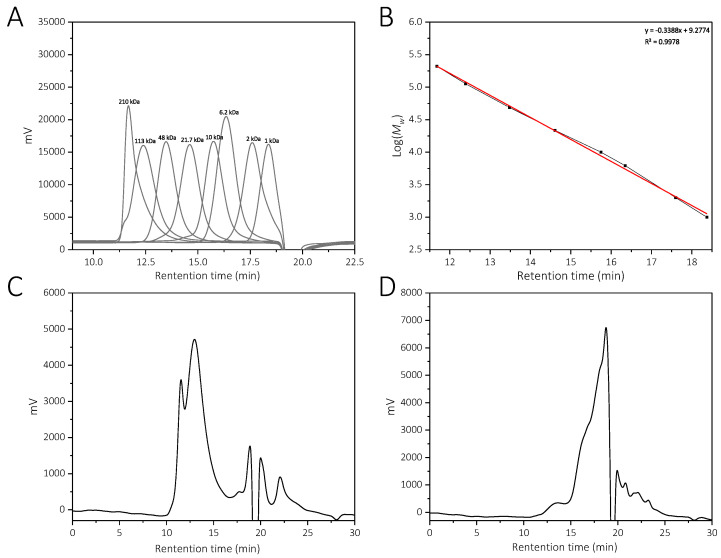
Relative molecular weight distribution of YCJP–1 and YCJP–2. (**A**) HPGPC profile of pullulan standards. (**B**) Linear regression equation obtained from pullulan standards. (**C**,**D**) HPGPC profiles for YCJP–1 and YCJP–2.

The molecular weight distributions of YCJP–1 and YCJP–2 were calculated according to the linear regression equation obtained from the pullulan standard (Figure 2A,B) [13]. Then, the relative molecular weights of YCJP–1 and YCJP–2 were determined using the HPGPC method, and the results are shown in Figure 2C,D. The results revealed that the molecular weights of YCJP–1 and YCJP–2 were distributed in 74,475–228,443 Da and 848–5810 Da.

#### 2.3.2. UV and FT-IR Analysis

**Figure 3:**
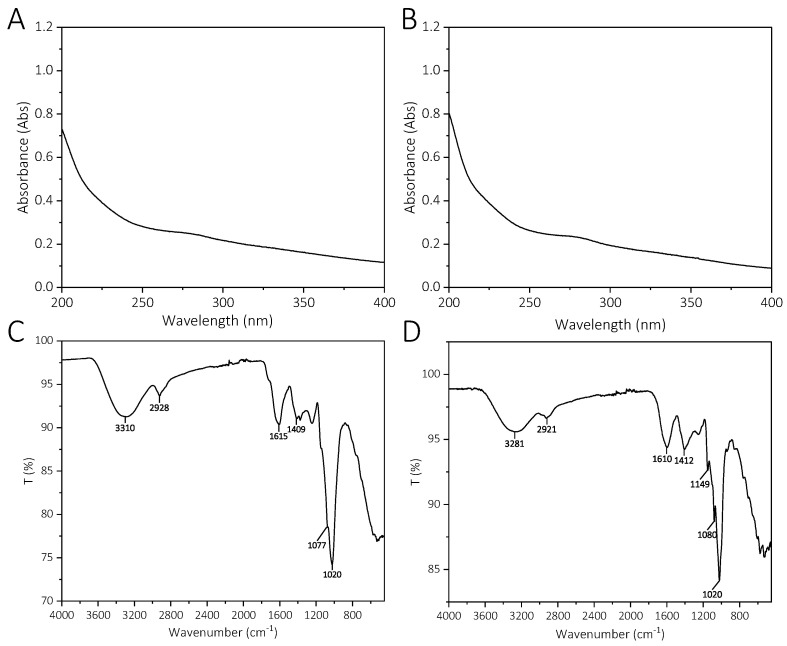
UV spectrum and FT-IR spectrum of YCJP–1 and YCJP–1. (**A**) UV spectra of YCJP–1. (**B**) UV spectra of YCJP–2. (**C**) FT-IR spectra of YCJP–1. (**D**) FT-IR spectrum of YCJP–2.

The UV spectrum of YCJP–1 and YCJP–2 is shown in Figure 3A,B. As suggested from the UV spectrum of YCJP–1 and YCJP–2, no significant absorptions were observed in 280 nm and 260 nm, indicating that YCJP–1 and YCJP–2 did not contain proteins and nucleic acids [14].

FT-IR was used to characterize the functional groups of YCJP–1 and YCJP–2. As shown in Figure 3C,D, both YCJP–1 and YCJP–2 possessed the characteristic absorption bands of polysaccharides [15]. For example, the strong absorption bands near 3300 cm^−1^ were attributed to the stretching vibration of O–H. The bands near 2920 cm^−1^ were caused by the bending vibration of C–H of methylene [16]. The bands at 1615/1610 cm^−1^ and 1409/1412 cm^−1^ were assigned to the asymmetric and symmetric stretching of COO^−^ of uronic acids [17]. The bands in the range of 1020–1149 cm^−1^ can be ascribed to the C‒O‒C asymmetric stretching vibration and the C‒O stretching vibration [18].

#### 2.3.3. Monosaccharide Composition Analysis

**Figure 4:**
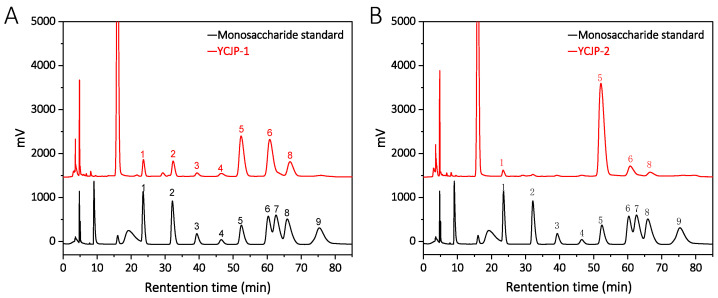
Monosaccharide composition analysis of YCJP–1 (**A**) and YCJP–2 (**B**) (1: mannose; 2: rhamnose; 3: glucuronic acid; 4: galacturonic acid; 5: glucose; 6: galactose; 7: xylose; 8: arabinose; 9: fucose).

The monosaccharide compositions of YCJP–1 and YCJP–2 were determined using the PMP precolumn derivatization method. As suggested from Figure 4A, we found that YCJP–1 was mainly composed of glucose, galactose, arabinose, mannose, and rhamnose, with small amount of uronic acids, including gluconic acid and galacturonic acid; the percentages of mannose, rhamnose, gluconic acid, galacturonic acid, glucose, galactose, and arabinose were calculated to be 7.06%, 8.86%, 2.34%, 2.77%, 37.85%, 41.03%, and 15.49%. In addition to YCJP–1, the monosaccharide composition of YCJP–2 was completely different than YCJP–1, which was mainly composed of glucose, with small amounts of mannose, galactose, and arabinose (Figure 4B). The percentages of mannose, glucose, galactose, and arabinose in YCJP–2 were calculated to be 2.46%, 84.12%, 9.87%, and 3.55%.

#### 2.3.4. SEM Analysis

**Figure 5:**
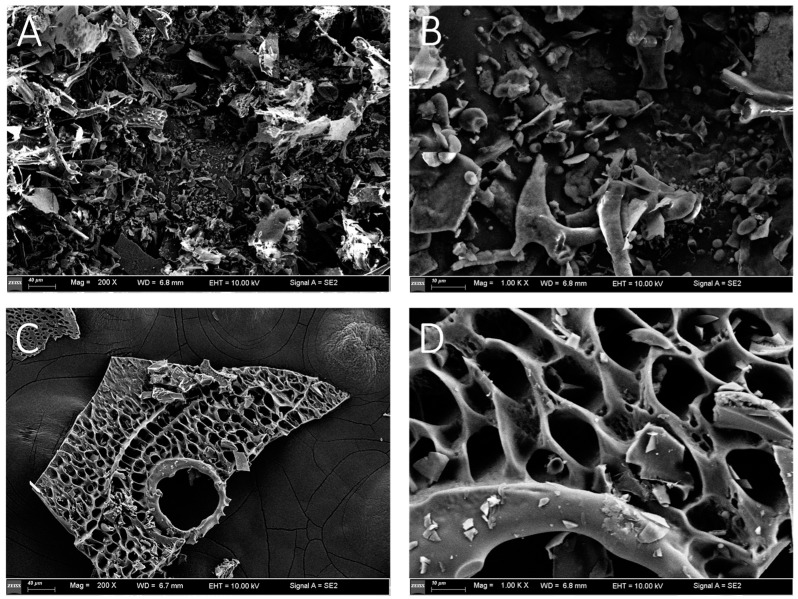
SEM images of YCJP–1 and YCJP–2 after 200- and 1000-times magnification. (**A**,**B**) Surface morphology of YCJP–1 (200× and 1000×). (**C**,**D**) Surface morphology of YCJP–2 (200× and 1000×).

The SEM images of YJCP-1 and YCJP–2 are shown in Figure 5. As suggested from Figure 5A, YCJP–1 mainly presented as irregular sheet-like structure, while YCJP–2 mainly presented as a sheet-like structure with regular mesh holes. These results might be due the fact that the monosaccharide composition of YCJP–1 was completely different from YCJP–2, finally leading to the physical and chemical properties of YCJP–1 and YCJP–2 being completely different. Similar results were reported by Saha et al. (2007), as revealed in [19].

### 2.4. Anti-Gastric Cancer Activity Evaluation of YCJP, YCJP–1, and YCJP–2

#### 2.4.1. Effects of YCJP, YCJP–1, and YCJP–2 on the Cell Viability of AGS and MGC-803 Cells

**Figure 6:**
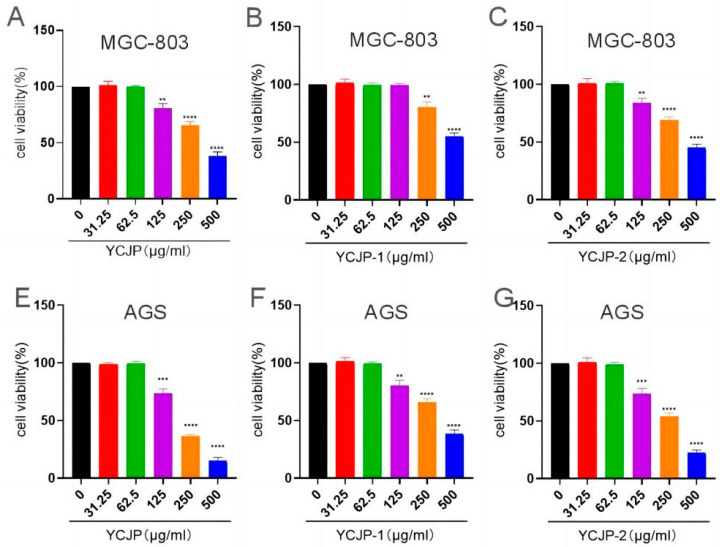
Inhibition effects of YCJP, YCJP–1, and YCJP–2 on MGC-803 and AGS cells. Data are shown as mean ± SD of at least three independent experiments. ** *p* < 0.01, *** *p* < 0.001, **** *p* < 0.0001 vs. the no drug treatment (0 μg/mL) group.

The effects of YCJP, YCJP–1, and YCJP–2 on the cell viability of AGS and MGC-803 cells were detected using the CCK-8 kit, and the results are shown in Figure 6. Notably, when compared to the control group, it was observed that YCJP, YCJP–1, and YCJP–2 could effectively inhibit the growth of both MGC-803 and AGS cells within the concentration range of 125–500 μg/mL (*p* < 0.01, *p* < 0.001). Moreover, comparing the inhibition effects of YCJP, YCJP–1, and YCJP–2 on the cell viability of MGC-803 cells and AGS cells revealed that YCJP, YCJP–1, and YCJP–2 exhibit stronger inhibitory effects on AGS cells than MGC-803 cells, indicating that AGS cells were more sensitive to YCJP, YCJP–1, and YCJP–2. In addition, when comparing the inhibition effects of YCJP–1 and YCJP–2 on MGC-803 cells and AGS cells, it was found that YCJP–2 possessed the more promising inhibitory effect on AGS cells and MGC-803 cells over YCJP–1; these results might be due to the fact that YJCP-2 possessed a lower molecular weight of and higher content of glucose than YCJP–1 [20]. In addition, when compared to other reported polysaccharides, YCJP–1 and YCJP–2 showed better anti-tumor activity. For example, the polysaccharide (SLP70-1) from sugarcane leaves showed almost no anti-tumor activity in the range of 25–400 μg/mL [21]. Moreover, Zhang et al. (2022) revealed that the polysaccharide from *Fomes officinalis* could effectively inhibit the proliferation of HepG2 cells in the concentration range of 50–800 μg/mL, and the maximum inhibition rate was only 59.86% [22].

#### 2.4.2. Effects of YCJP, YCJP–1, and YCJP–2 on the Colony and Proliferation of AGS and MGC-803 Cells

**Figure 7:**
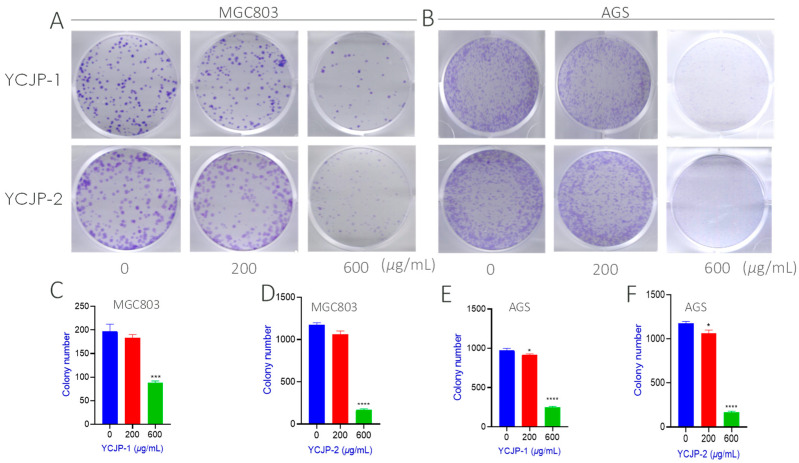
Inhibition effects of YCJP, YCJP–1, and YCJP–2 on the colony formation efficiency of MGC-803 and AGS cells. (**A**,**B**) Representative images of YCJP–1 and YCJP–2 on the colony formation efficiency of MGC-803 and AGS cells. (**C**–**F**) Quantitative analysis results of YCJP–1 and YCJP–2 on the colony formation efficiency of MGC-803 and AGS cells. All values are expressed as the mean ± SD of at least three independent experiments. * *p* < 0.05, *** *p* < 0.001, **** *p* < 0.0001 vs. the no drug treatment (0 μg/mL) group.

Colony-forming efficiency refers to the cell vaccination survival rate, which represents the number of cells that survived and formed clones after inoculation [23]. In order to further evaluate the anti-cloning effects of YCJP–1 and YCJP–2 on tumor cells, the colony-forming assay was carried out in this study. As suggested from Figure 7A,B, it was found that the purple crystal in the YCJP–1 and YCJP–2 treated group was obviously decreased when compared to the not polysaccharide-treated group, indicating that YCJP–1 and YCJP–2 could effectively inhibit the proliferation of MGC-803 and AGS cells. The inhibitory effects were further quantified, and the results are shown in Figure 7C–F. As expected, both YCJP–1 and YCJP–2 could effectively inhibit the colony number of MGC-803 cells (*p* < 0.001) with a concentration of 600 μg/mL. As shown in Figure 7C,D, the colony numbers of MGC-803 cells in the YCJP–1 and YCJP–2 treated groups were 50 and 70, which were 4.5 and 3 times lower than that of the vehicle control group (0 μg/mL) (Figure 7C,D). In addition, for ASG cells, similar results were observed, further confirming that YCJP–1 and YCJP–2 possess prominent anti-gastric cancer activity via inhibiting the colony and proliferation of cancer cells.

### 2.5. Discussion

It was reported that the different polysaccharides have different anti-tumor effects, and the anti-rumor effects of polysaccharides are closely related to the chemical structure of polysaccharides, including their molecular weight, monosaccharide unit and content, the type and configuration of glycosidic bonds, and the branching degree [20]. Among these structural factors, the type and content of monosaccharides are considered to be two of the important factors affecting the anti-tumor activity of polysaccharides, especially glucose. It was reported that glucan is the basic structural unit of many anticancer polysaccharides. For examples, glucan from *Lentinus edodes* (Berk.) Pegler can block colon cancer cells’ cycle by inhibiting cyclin production, inducing cellular stress-responsive pathways and the transcription of metastasis suppressor genes [24]. Awadasseid et al. (2017) revealed that the water-soluble polysaccharide from *Coriolus versicolar*, which is mainly composed of glucan, has significant anticancer activity [25]. Our results also revealed that the content of glucose is one of the key factors of the antitumor activity of YCJP–1 and YCJP–2, and the higher anti-rumor activity of YCJP–2 can be ascribed to its higher content of glucose. In addition to monosaccharide content and type, molecular weight is another important factor impacting the anti-rumor activity of polysaccharides [24]. It was reported that few polysaccharides with low molecular weights (<5 kDa), extended from *Sclerotium rolfsii* and the basidio mycete *Schizophyllum commune*, exert a significant anticancer activity [26,27], which might be due to the fact that relatively lower molecular weight polysaccharides more easily interact with the proteins on the cell membrane surface or are more easily taken in by cancer cells. Similarly, our results also confirmed this conclusion, which showed that the molecular weight of YCJP–2 (<5 kDa) is much smaller than that of YCJP–1. Thus, based on the results described above, we can preliminarily draw the conclusion that the higher anti-tumor activity of YCJP–2 is closely related to its higher content of glucose and lower molecular weight.

## 3. Materials and Methods

### 3.1. Materials and Reagents

Dried *Chloranthus japonicus* herbs were obtained from Nanning city, Guangxi province, China, and identified by professor Depo Yang of Sun Yat-sen University (Guangzhou, China). The specimens of *C. japonicus* herbs were stored in the Laboratory of Pharmacy and Natural Medicinal Chemistry, School of Pharmacy, Sun Yat-sen University (No. 20220922). Monosaccharide standards (glucose, mannose, rhamnose, glucuronic acid, galacturonic acid, galactose, xylose, arabinose, fucose) and pullulan standards were purchased from Aladdin Biochemical Technology Co., Ltd. (Shanghai, China) and Zhenzhun Biotechnology Co., Ltd. (Shanghai, China). BCA protein assay kit was obtained from Beyotime Biotechnology Co., Ltd. (Beijing, China). Penicillin/streptomycin and fetal bovine serum (FBS) were obtained from Gibco. All the other chemical compounds used for testing were of analytical grade.

### 3.2. Preparation of Crude C. japonicus Polysaccharide (YCJP)

**Figure 8:**
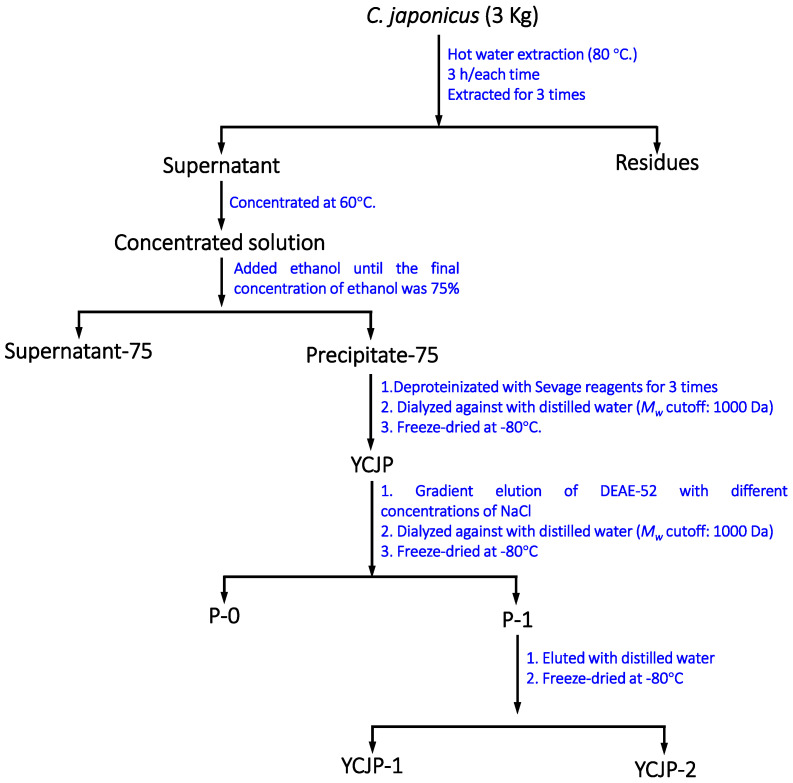
Extraction products of YCJP, and isolation products of YCJP–1 and YCJP–2.

The dried *C. japonicus* herbs (3 kg) were crushed to a powder and immersed in 20 L of anhydrous ethanol for 3 weeks to remove the small-molecule substances. After that, the *C. japonicus* herb powder was further re-dried and extracted with 20 L of distilled water at 95 °C for 2 h using an extraction tank (50 L). The extraction process was repeated twice, and the decoction was combined and concentrated to one tenth of the original volume using a rotary evaporator at 60 °C. Subsequently, anhydrous ethanol was added into the concentrated water extracts of *C. japonicus* herbs until the final concentration of ethanol was 75%. The mixture was stored at 4 °C overnight. The precipitate was collected the next day and redissolved in 2 L of distilled water. After that, the polysaccharide solution was further deproteinated using Sevage reagent (chloroform and n-butanol = 4:1) three times. The deproteinization solution was then concentrated and dialyzed with running water for 48 h (*M_w_* cut off: 3500 Da). Then, the dialysate was collected, concentrated, and freeze-dried at −80 °C to obtain the crude polysaccharide and labelled as YCJP. The detailed extraction process of YCJP–1 is shown in Figure 8.

The contents of carbohydrate polymers, uronic acid, and proteins in YCJP were determined using the phenol-sulfuric acid method, M-hydroxybiphenyl method, and BCA kit, respectively [28,29,30].

### 3.3. Isolation and Purification of YCJP

The obtained YCJP was first purified using a DEAE-52 column and Sepharose CL-6B column. Briefly, 20 mg/mL of YCJP was fractioned on the DEAE-52 column [31] and eluted with different concentrations of sodium chloride solution ranging from 0 to 1.0 M. The eluents eluted with different concentrations of sodium chloride solution were collected using an auto fraction collector. After that, the different eluents were further concentrated, dialyzed (Mw cut off: 1000 Da), and freeze-dried. The freeze-dried samples were further isolated using a Sepharose CL-6B column. The distilled water was used as the eluted solution and the eluent was collected and freeze-dried to offer the purified polysaccharides, which were labelled as YCJP–1 and YCJP–2, respectively. The contents of polysaccharides and proteins in YCJP–1 and YCJP–2 were determined using the methods described in Section 2.2.

### 3.4. Structural Features of YCJP–1 and YCJP–2

#### 3.4.1. Relative Molecular Weight Distribution Determination

The relative molecular weight distributions of YCJP–1 and YCJP–2 were analyzed using a high-performance liquid gel permeation (HPGCP) system coupled with a refractive index detector (RID) [32]. Briefly, 5 mg of dried sample was dissolved in 1 mL of distilled water and filtered using 0.22 μM of filter membrane. After that, the filtered YCJP–1 and YCJP–2 solutions were further subjected to Agilent 1200 high-performance liquid chromatography (Agilent, Santa Clara, CA, USA). The chromatographic conditions used in this assay were as follows.

Chromatographic column: TSK-GEL G3000 PW_XL_ column (Tosoh Biosep, Yamaguchi, Japan). The mobile phase was 0.02 M potassium dihydrogen phosphate solution, and the flow rate was set as 0.5 mL/min. The column temperature was 35 °C and the injection volume was 20 μL.

#### 3.4.2. UV and FT-IR Spectra Analysis

The UV and FT-IR spectra of YCJP–1 and YCJP–2 were recorded according to the methods of previous reports [33].

#### 3.4.3. Monosaccharide Composition Analysis

The 1-phenyl-3-methyl-5-pyrazolone (PMP) pre-column derivatization method was used to determine the monosaccharide composition of YCJP–1 and YCJP–2 [34]. In brief, 4 mg of sample (YCJP–1 or YCJP–2) was dissolved in 2 mL of trifluoroacetic acid (TFA) and reacted at 120 °C for 4 h. After that, the reacted solution was co-concentrated with absolute methanol 4 times to remove TFA completely. Distilled water (1 mL) was then added into the hydrolyzed products and then reacted with the PMP reagent. After the reaction, the PMP-derivatives of the sample were analyzed using an Agilent 1200 HPLC system (Agilent, Santa Clara, CA, USA) coupled with an ultraviolet detector. The chromatographic conditions were as follows. The column was an Ultimate XB-C18 column (5 μm, 4.6 × 250 mm) with a flow rate of 1 mL/min. The mobile phase was acetonitrile and a phosphate buffer solution (17:83, *v*/*v*, pH = 6.9). The detected wavelength was 250 nm, and the injection volume was 20 μL.

#### 3.4.4. Scanning Electron Microscopy (SEM) Analysis

The SEM images of YCJP–1 and YCJP–2 were recorded using a FE-SEM at an accelerating voltage of 15 kV. The sample was prepared according to the methods reported by Zhang et al. (2024) [35].

### 3.5. Anti-Gastric Cancer Activity Evaluation of YCJP, YCJP–1, and YCJP–2

#### 3.5.1. Cell Culture

Human gastric cancer cell lines MGC-803 and AGS were offered by Shanghai Institute for Biological Science, Chinese Academy of Sciences. Cells were then cultured in RPMI-1640 medium supplemented with 10% (*v*/*v*) fetal bovine serum and 100 U/mL penicillin/streptomycin under a water-saturated atmosphere of 95% air and 5% CO_2_.

#### 3.5.2. CCK-8 Assay

The CCK-8 assay was used to evaluate the anti-gastric activities of YCJP, YCJP–1, and YCJP–2. In brief, cells (AGS or MGC-803) were seeded in 96-well plates at a density of 5 × 10^3^ cells per well and further incubated at 37 °C for 24 h. Then, different concentrations of YCJP, YCJP–1, and YCJP–2 (31.3, 62.5, 125, 250, and 500 μg/mL) were added into the 96-well plates and further incubated with cancer cells for 48 h. Subsequently, CCK-8 solution (10 µL) was added into the 96-well plates and further incubated at 37 °C for 2 h. After incubation, the absorbances in each well were then recorded using a multimode reader at 450 nm. The effects of YCJP, YCIP-1, and YCJP–2 on the cell viability of AGS and MGC-803 were then calculated according to the obtained absorbances.

#### 3.5.3. Colony-Forming Assay

The inhibitory effects of YCJP, YCJP–1, YCJP–2 on the proliferation ability of AGS and MGC-803 cell were further evaluated using colony-forming assay. In brief, AGS and MGC-803 cells were first seeded into 6-well plates at a density of 500 cells/well and 800 cells/well, then incubated at 37 °C for 24 h. After incubation, different concentrations of YCJP–1 and YCJP–2 were added (0, 200, and 600 μg/mL) and the cells were further incubated at 37 °C for 12 days. Subsequently, the cells were fixed by PFA (1%, 1mL) for 15 min. After that, the PFA was discarded, and 1 mL of crystal violet staining solution was added and incubated for another 30 min. Then, the cells were further washed with sterile water 3 times to remove the residual crystal violet staining solution. Finally, the cells were imaged with a microscope and the effects of YCJP–1 and YCJP–2 on the colony number of cancer cells were further quantified using an Image J 22 software.

### 3.6. Statistical Analysis

Data in this study were analyzed using GraphPad prism 8.0 software and presented as the mean ± SD. Differences between control group and experiment group were analyzed using two-tailed Student’s *t*-test. Probabilities (*p*) less than 0.05 were determined to be significant by analysis of variance (ANOVA).

## 4. Conclusions

In this present study, two novel heteropolysaccharides, named YCJP–1 and YCJP–2, were obtained from *C. japonicus*. The structural features of YCJP–1 were distinctly different from YCJP–2. YCP-1 was mainly composed of glucose, galactose, arabinose, mannose, rhamnose, and small amounts of uronic acids (gluconic acid and galacturonic acid), with the relative molecular weights mainly distributed in the range of 74,475–228,443 Da. YCJP–2 was mainly composed of glucose and small amounts of mannose, galactose, and arabinose, with the relative molecular weights mainly distilled in the range of 848–5810 Da. An in vitro assay further demonstrated that both YCJP–1 and YCJP–2 possess prominent anti-gastric cancer activity via inhibiting the proliferation and colony formation of MGC-803 and AGS cells. The difference in anti-tumor activity between YCJP–1 and YCJP–2 may be due to the distinct chemical properties between the isolated polysaccharides, and to the fact that YCJP–2 has a higher glucose content and a relatively lower molecular weight compared to YCJP–1. Overall, our study confirmed that the obtained polysaccharides, YCJP–1 and YCJP–2, could potentially serve as the new bioactive molecules for the development of an anti-gastric cancer agent. In addition, our results further confirmed that the relatively lower molecular weights and higher glucose contents were the key factors of the anti-tumor activity of polysaccharides, which provides a theoretical basis for the development of polysaccharide anti-tumor drugs. Although our research has preliminarily revealed that YCJP–1 and YCJP–2 possess prominent anti-proliferative activity on MGC-803 cells and AGS cells, further analysis of their purification, structure, in vivo anti-tumor activity, and molecular mechanism is needed for understanding the relationship between activity and polysaccharide structure.

## Data Availability

Data are available upon request from the corresponding author.
